# Pneumonic-type invasive mucinous adenocarcinoma and infectious pneumonia: clinical and CT imaging analysis from multiple centers

**DOI:** 10.1186/s12890-022-02268-5

**Published:** 2022-12-03

**Authors:** Shuai Zhang, Xinxin Yu, Yong Huang, Pei Nie, Yan Deng, Ning Mao, Sha Li, Baosen Zhu, Li Wang, Bo Wang, Ximing Wang

**Affiliations:** 1 School of Medicine, Shandong First Medical University, No. 6699, Qingdao Road, Jinan, 250024 Shandong China; 2 Shandong Provincial Hospital, Shandong University, No. 324, Jingwu Road, Jinan, 250021 Shandong China; 3 Shandong Cancer Hospital and Institute, Shandong First Medical University and Shandong Academy of Medical Sciences, No. 440, Jiyan Road, Jinan, 250117 Shandong China; 4 Department of Radiology, The Affiliated Hospital of Qingdao University, No. 16, Jiangsu Road, Qingdao, 266000 Shandong China; 5 Department of Radiology, Qilu Hospital, Shandong University, No. 107, Wenhua West Road, Jinan, 250012 Shandong China; 6 Department of Radiology, Yantai Yuhuangding Hospital, Affiliated Hospital of Qingdao University, No. 20, Yuhuangding East Road, Yantai, 164000 Shandong China; 7 Physical Examination Center, Shandong Provincial Hospital Affiliated to Shandong First Medical University, No. 324, Jingwu Road, Jinan, 250021 Shandong China; 8 Department of Anesthesiology, Shandong Provincial Hospital, Shandong University, No. 324, Jingwu Road, Jinan, 250021 Shandong China; 9 Department of Anesthesiology, Shandong Provincial Hospital Affiliated to Shandong First Medical University, No. 324, Jingwu Road, Jinan, 250021 Shandong China; 10 Department of Radiology, Shandong Provincial Hospital Affiliated to Shandong First Medical University, Shandong University, No. 324, Jingwu Road, Jinan, 250021 Shandong China

**Keywords:** Invasive mucinous adenocarcinoma, Infectious pneumonia, Computed tomography, Lung disease

## Abstract

**Background:**

Pneumonic-type invasive mucinous adenocarcinoma (IMA) was often misdiagnosed as pneumonia in clinic. However, the treatment of these two diseases is different.

**Methods:**

A total of 341 patients with pneumonic-type IMA (n = 134) and infectious pneumonia (n = 207) were retrospectively enrolled from January 2017 to January 2022 at six centers. Detailed clinical and CT imaging characteristics of two groups were analyzed and the characteristics between the two groups were compared by χ^2^ test and Student’s t test. The multivariate logistic regression analysis was performed to identify independent predictors. Receiver operating characteristic curve analysis was used to determine the diagnostic performance of different variables.

**Results:**

A significant difference was found in age, fever, no symptoms, elevation of white blood cell count and C-reactive protein level, family history of cancer, air bronchogram, interlobular fissure bulging, satellite lesions, and CT attenuation value (all p < 0.05). Age (odds ratio [OR], 1.034; 95% confidence interval [CI] 1.008–1.061, *p* = 0.010), elevation of C-reactive protein level (OR, 0.439; 95% CI 0.217–0.890, *p* = 0.022), fever (OR, 0.104; 95% CI 0.048–0.229, *p* < 0.001), family history of cancer (OR, 5.123; 95% CI 1.981–13.245, *p* = 0.001), air space (OR, 6.587; 95% CI 3.319–13.073, *p* < 0.001), and CT attenuation value (OR, 0.840; 95% CI 0.796–0.886, *p* < 0.001) were the independent predictors of pneumonic-type IMA, with an area under the curve of 0.893 (95% CI 0.856–0.924, *p* < 0.001).

**Conclusion:**

Detailed evaluation of clinical and CT imaging characteristics is useful for differentiating pneumonic-type IMA and infectious pneumonia.

## Introduction

Lung cancer is currently the leading cause of cancer incidence and mortality worldwide, and lung adenocarcinoma is the main subtype of lung cancer [1, 2]. Compared with other lung adenocarcinoma subtypes, invasive mucinous adenocarcinoma (IMA), a rare and distinct subtype of lung adenocarcinoma, has significantly worse prognosis and is more prone to lung metastasis [3, 4]. IMA characterized by consolidations opacities on CT imaging mimicking pneumonia was recognized as pneumonic-type IMA [5]. In view of the overlapping clinical symptoms and radiological features, this type of pulmonary IMA is often misdiagnosed as infectious pneumonia, resulting in a delay in definitive management and an increased risk of death in the affected patients [6].

Several studies have assessed the clinical, imaging, and pathological features of pneumonic-type lung IMA [5, 7], however, they didn’t investigate to distinguish it from infectious pneumonia. Jung et al. [8] and Kim et al. [9] previously distinguished between mucinous bronchioloalveolar carcinoma, redefined as IMA nowadays, and infectious pneumonia by CT characteristics. Given the low incidence of IMA, accounting for 2% to 5% of all adenocarcinomas [10], most studies about pneumonic-type IMA were with small samples and without sufficient clinical information. Therefore, comprehensive clinical and imaging studies with larger samples to differentiate pneumonic-type IMA and infectious pneumonia are imminent.

The purpose of this study was to evaluate the clinical and CT imaging features between pneumonic-type IMA and infectious pneumonia.

## Materials and methods

### Patients

Institutional review board approval was obtained, and patient informed consent was waived due to the retrospective nature of this study.

**Fig. 1:**
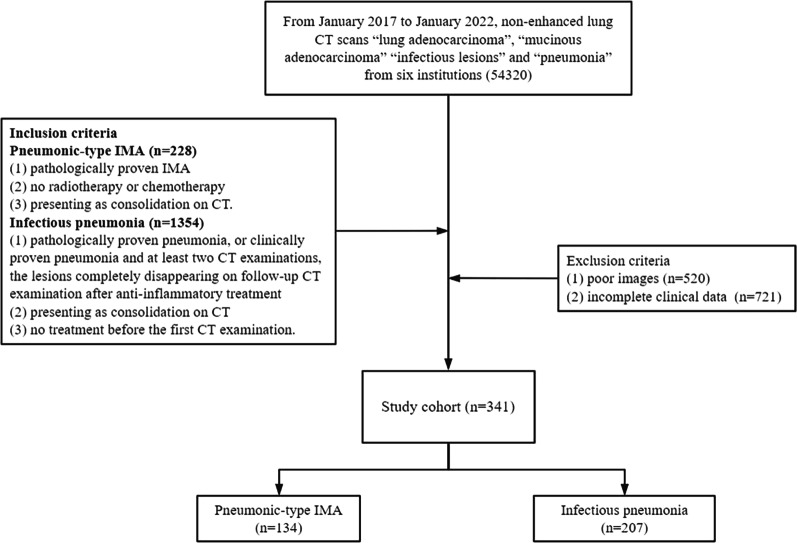
Patient flow chart and patient selection

We searched the radiology reports using the terms “lung adenocarcinoma”, “mucinous adenocarcinoma” “infectious lesions” and “pneumonia” on non-enhanced lung CT scans from January 2017 to January 2022 at six institutions (Affiliated Shandong Provincial Hospital of Shandong First Medical University; Shandong Province Yuhuangding Hospital; Shandong Tumor Hospital; Affiliated Hospital of Jining Medical University; Affiliated Hospital of Qingdao University; Qilu Hospital of Shandong University). Inclusion criteria for pneumonic-type IMA were: (1) pathologically proven IMA, (2) no radiotherapy or chemotherapy, and (3) presenting as consolidation on CT. Inclusion criteria for infectious pneumonia were: (1) pathologically proven pneumonia, or (2) clinically proven pneumonia and at least two CT examinations, the lesions completely disappearing on follow-up CT examination after anti-inflammatory treatment, (3) presenting as consolidation on CT, and (4) no treatment before the first CT examination. Common exclusion criteria for both diseases were as follows: (1) poor images, and (2) incomplete clinical data. If a patient had multiple lesions, then a single largest lesion was analyzed each patient. And if a patient had at least two CT examinations, the first CT examination was used for analysis. Two radiologists (S.Z. and X.X.Y., with 7 and 10 years of experience in lung radiology, respectively) made consensus decisions on correlation. To avoid recall bias, these two radiologists were not involved in CT image analysis evaluation. Flowchart for selecting the study population is shown in Fig. 1.

Demographic and clinical information including age, sex, smoking, cough, sputum, fever, no symptoms, laboratory results (elevation of white blood cell count [> 10 × 10^9^/L] and C-reactive protein level [> 10 mg/L]), family history of cancer was collected from medical record.

### CT protocols

All non-enhanced CT images were obtained on the multidetector CT scanners (Ingenuity CT, Philips; Brilliance iCT, Philips; Somatom Force, Siemens Healthcare; Somatom Definition Flash, Siemens Healthcare; Somatom Definition AS, Siemens; Optima CT660, GE Healthcare; Discovery 750, GE Healthcare). The chest CT scanning parameters were the following: tube voltage of 120 kVp, pitch of 0.8–1.0, 250–400 mA (using automatic tube current modulation technique) tube current, a matrix of 512 × 512, reconstructed slice thickness of 1 mm, reconstructed slice interval of 1 mm, rotation time of 500–600 ms. Non-enhanced CT scanning was performed with coverage from the thoracic inlet to the lung base in the supine position.

### Image analysis

All images were independently evaluated by two radiologists (S.L. and X.M.W.) with more than 10-year’ experience in chest imaging, both of whom were blinded to patient clinical information with any disagreement in assessment resolved by consensus.

The pneumonia type IMA is defined as consolidation along the lung lobe or segment without definite shape on CT [5]. The assessments of CT features of lesions including location (unilateral or bilateral), margin (well-defined or ill-defined), air bronchogram (absence, regular, or irregular), interlobular fissure bulging (absent or present), air space (absent or present), satellite lesions (absent or present), pleural effusion (absent or present), lymphadenopathy (absent or present), and CT attenuation value were obtained by using post-processing workstation (Syngo.via, Siemens Force, Germany). Among them, air bronchogram was defined as air-filled bronchi within lesions, and irregular air bronchogram appeared as dilatation, stiffness, or narrowing of bronchi. Lymphadenopathy was defined as hilar or mediastinal lymph nodes > 1 cm in short axis diameter. On non-enhanced CT images, three circular regions of interests (ROIs) were selected at the maximum cross-sectional area of the lesions, avoiding vessels, bronchi, and air space. Then, we measured the non-enhanced CT values of the ROIs and calculated the mean value. All CT attenuation measurements are reported in HU. Inter-observer agreement in evaluation of CT image was calculated using the intraclass correlation coefficient and kappa statistics.

### Statistical analysis

Statistical analysis was performed using SPSS (version 22.0, IBM) and R statistical software (version 3.3.3, https://www.r-project.org). Continuous variables were described as mean ± standard deviation, whereas categorical variables were expressed as percentage. Clinical information and CT image characteristics were compared between pneumonic-type IMA and infectious pneumonia by using Student’s t test and χ^2^ test. Moreover, multivariate logistic regression analysis with generalized estimating equation correction was performed to calculate odds ratio (OR) and the corresponding 95% confidence interval (CI) of the independent predictors. Receiver operating characteristic (ROC) curve analysis was performed to analyze statistically significant variables in differentiating pneumonic-type IMA and infectious pneumonia. The diagnostic performance was assessed by the area under the curve (AUC), sensitivity, specificity, and accuracy. Differences in the AUC values were estimated using the Delong test. A *p* < 0.05 was considered statistically significant. The logistic regression prediction formula was defined as: P(z) = 1/(1 + e^−z^), z = θX + b, where X is the characteristics variable, θ is the weight variable, and b is the intercept.

## Results

### Study population

Five hundred and twenty patients with poor images were excluded, and seven hundred and twenty-one patients without complete clinical data were excluded. A total of 341 patients (mean age ± standard deviation, 66 ± 42 years; 12 men) with pneumonic-type IMA (n = 134) and infectious pneumonia (n = 207) from six institutions were enrolled in this study. In the pathological proven infectious pneumonia (n = 76) and pneumonia-type IMA (n = 134), 42 patients with infectious pneumonia and 107 patients with pneumonia-type IMA were determined by surgery, and the remainders were determined by biopsy. The clinical data are summarized in Table 1.

**Table 1:** The clinical characteristics between pneumonic-type IMA and infectious pneumonia

Characteristics	Total (n = 341)	Pneumonic-type IMA (n = 134)	Infectious pneumonia (n = 207)	*p* value
Age, years	61.5 ± 13.5	63.8 ± 10.2	59.9 ± 15.0	0.009
Sex, male	190 (55.7)	70 (52.2)	120 (58.0)	0.298
Smoking	126 (37.0)	49 (36.6)	77 (37.2)	0.906
Cough	247 (72.4)	102 (76.1)	145 (70.0)	0.220
Sputum	240 (70.4)	101 (75.4)	139 (67.1)	0.104
Fever	133 (39.0)	19 (14.2)	114 (55.1)	< 0.001
No symptoms	42 (12.3)	24 (17.9)	18 (8.7)	0.011
Elevation of white blood cell count	99 (29.0)	27 (20.1)	72 (34.8)	0.004
Elevation of C-reactive protein level	199 (58.4)	56 (41.8)	143 (69.1)	< 0.001
Family history of cancer	44 (12.9)	24 (17.9)	20 (9.7)	0.026

Continuous variables are described as mean ± standard deviation, and categorical variables are presented as numbers (%)

### Comparison of clinical data between pneumonic-type IMA and infectious pneumonia

As shown in Table 1, compared with patients with infectious pneumonia, those with pneumonic-type IMA tended to be older (63.8 ± 10.2 years vs. 59.9 ± 15.0 years; *p* < 0.001). The patients with pneumonic-type IMA showed a higher prevalence of no symptoms (17.9% vs. 8.7%, *p* = 0.011) and family history of cancer (17.9% vs. 9.7%, *p* = 0.026) than those with infectious pneumonia. Fever (55.1% vs. 14.2%, *p* < 0.001), elevation of white blood cell count (34.8% vs. 20.1%, *p* = 0.004), and elevation of C-reactive protein level (69.1% vs. 41.8%, *p* < 0.001) were more commonly observed in patients with infectious pneumonia than those with pneumonic-type IMA. No significant difference was founded in other clinical information between two groups (*p* > 0.05).

### Comparison of CT imaging features between pneumonic-type IMA and infectious pneumonia

**Fig. 2:**
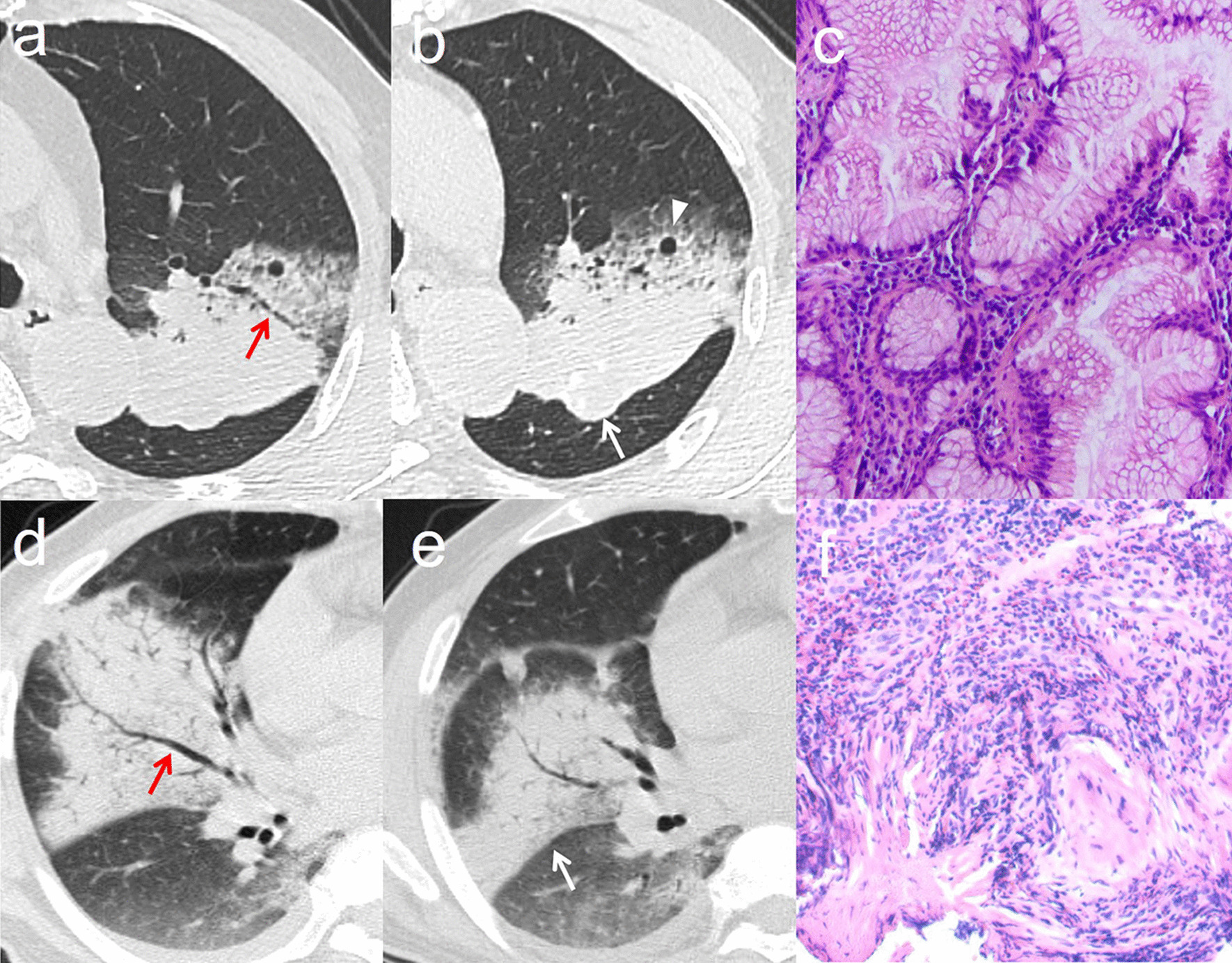
Representative images of CT imaging characteristics. Non-enhanced CT images (**a**, **b**) of lung window from a 69-year-old-male patient indicate the consolidation with irregular air bronchogram (red arrow), air space (white arrowhead), and interlobular fissure bulging (white arrow) in the left upper lobe. Pathology (**c**) confirmed invasive mucinous adenocarcinoma (hematoxylin and eosin staining, × 200). Non-enhanced CT images (**d**, **e**) of lung window from a 62-year-old-male patient indicate the consolidation with regular air bronchogram (red arrow) and no interlobular fissure bulging (white arrow) in the right middle lobe. Pathology (**f**) confirmed infectious pneumonia (hematoxylin and eosin staining, × 200)

The detailed CT characteristics of two groups are presented in Table 2. Irregular Air bronchogram (50.7% vs. 37.7%, *p* = 0.016), interlobular fissure bulging (18.7% vs. 9.7%, *p* = 0.017), air space (65.7% vs. 30.0%, p < 0.001), and satellite lesions (19.4% vs. 11.6%, *p* = 0.046) were frequently present in pneumonic-type IMA than infectious pneumonia (Fig. 2). The pneumonic-type IMA had lower CT attenuation value (25.9 ± 7.1 HU vs. 32.3 ± 5.1 HU, *p* < 0.001) than infectious pneumonia. However, no significant differences were observed in other CT features between two groups (*p* > 0.05).

**Table 2:** Comparison of CT imaging features between pneumonic-type IMA and infectious pneumonia

Characteristics	Pneumonic-type IMA (n = 134)	Infectious pneumonia (n = 207)	*p* value
Location			0.176
Unilateral	90 (67.2)	124 (59.9)	
Bilateral	44 (32.8)	83 (40.1)	
Margin			0.159
Well-defined	25 (18.7)	27 (13.0)	
Ill-defined	109 (81.3)	180 (87.0)	
Air bronchogram			0.016
Absent	25 (18.7)	34 (16.4)	
Regular	41 (30.6)	95 (45.9)	
Irregular	68 (50.7)	78 (37.7)	
Interlobular fissure bulging			0.017
Absent	109 (81.3)	187 (90.3)	
Present	25 (18.7)	20 (9.7)	
Air space			< 0.001
Absent	46 (34.3)	145 (70)	
Present	88 (65.7)	62 (30.0)	
Satellite lesions			0.046
Absent	108 (80.6)	183 (88.4)	
Present	26 (19.4)	24 (11.6)	
Pleural effusion			0.234
Absent	80 (59.7)	110 (53.1)	
Present	54 (40.3)	97 (46.9)	
Lymphadenopathy			0.710
Absent	90 (67.2)	143 (69.1)	
Present	44 (32.8)	64 (30.9)	
CT attenuation value, HU	25.9 ± 7.1	32.3 ± 5.1	< 0.001

Continuous variables are described as mean ± standard deviation, and categorical variables are presented as numbers (%)

### Multivariate logistic regression analysis

**Fig. 3:**
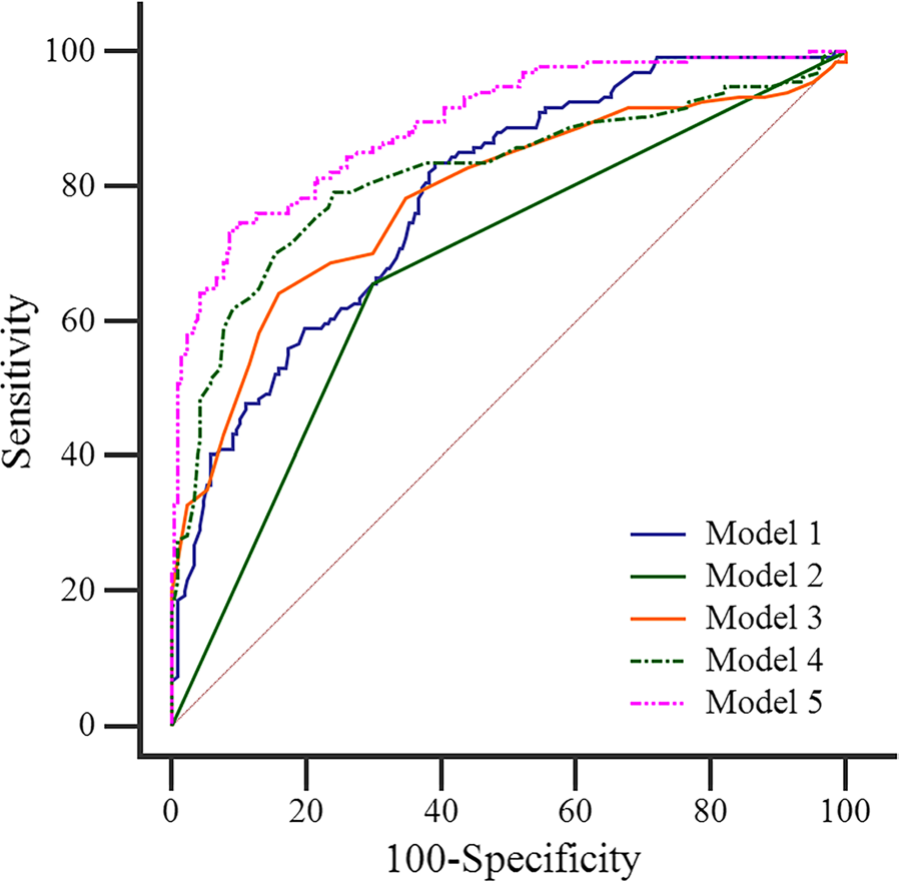
ROC analysis in differentiating between pneumonic-type invasive mucinous adenocarcinoma and infectious pneumonia. Model 1: age, elevation of C-reactive protein level, fever and family history of cancer; Model 2: air space; Model 3: CT attenuation value; Model 4: model 2 + model 3; Model 5: model 1 + model 4

By multivariate logistic regression analysis, age (OR, 1.034; 95% CI 1.008–1.061, *p* = 0.010), elevation of C-reactive protein level (OR, 0.439; 95% CI 0.217–0.890, *p* = 0.022), fever (OR, 0.104; 95% CI 0.048–0.229, *p* < 0.001), family history of cancer (OR, 5.123; 95% CI 1.981–13.245, *p* = 0.001), air space (OR, 6.587; 95% CI 3.319–13.073, *p* < 0.001), CT attenuation value (OR, 0.840; 95% CI 0.796–0.886, *p* < 0.001) were the independent predictors of pneumonic-type IMA (Fig. 3).

### Assessment of diagnostic performance of models

**Fig. 4:**
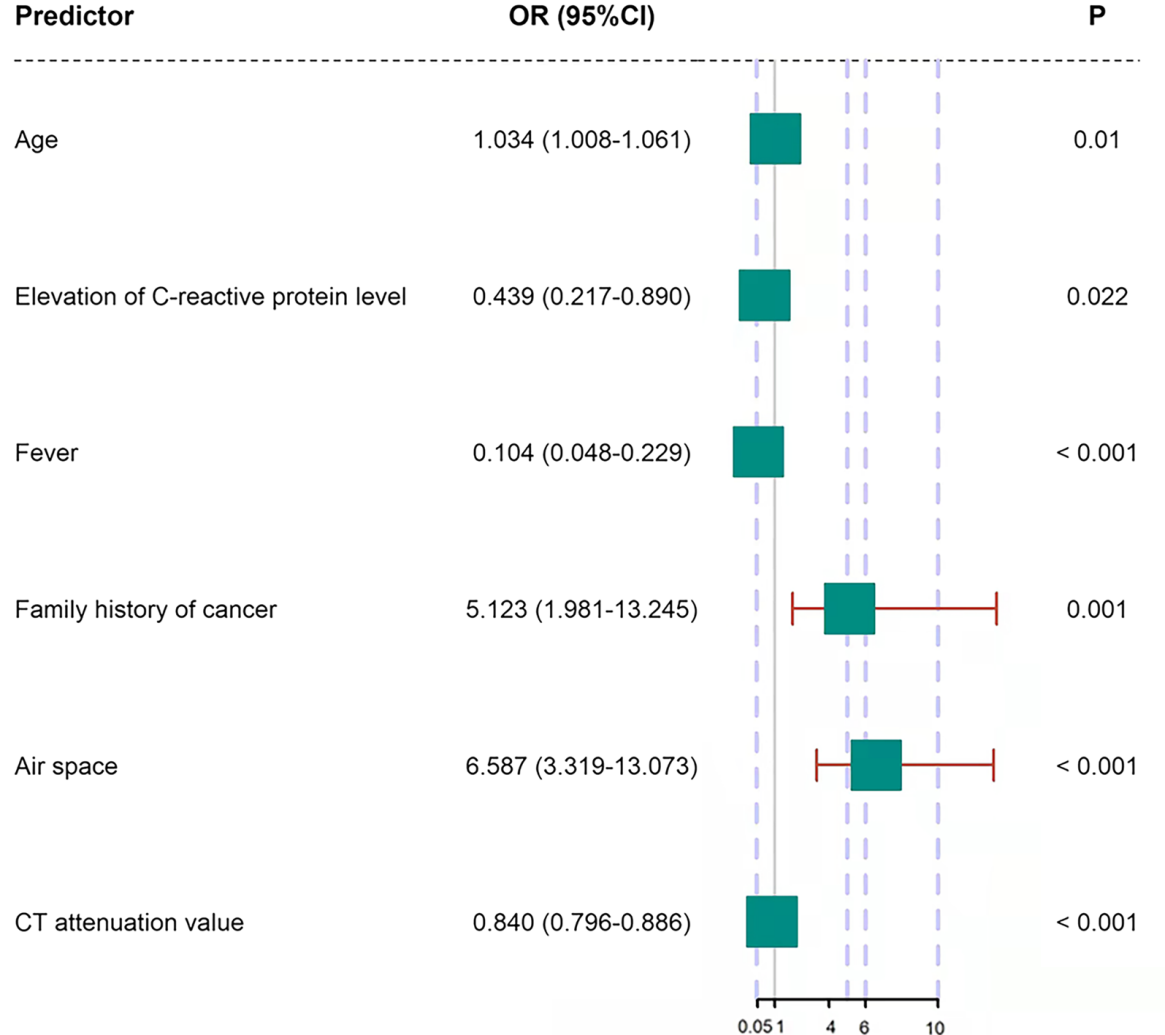
The independent predictors of pneumonic-type invasive mucinous adenocarcinoma

For differentiating between pneumonic-type IMA and infectious pneumonia using ROC analysis, we analyzed the combined clinical variables of age, elevation of C-reactive protein level, fever and family history of cancer (model 1), air space (model 2), CT attenuation value (model 3), combination of air space and CT attenuation value (model 4), and combination of clinical variables of age, elevation of C-reactive protein level, fever, family history of cancer, air space and CT attenuation value (model 5). The diagnostic performances of different models and their corresponding AUCs are shown in Table 3, and the ROC curves for different models are shown in Fig. 4. For model 3, the best cut-off of CT attenuation value was 27.0 HU. Model 5 had better diagnostic performance with an AUC of 0.893 (95% CI 0.856–0.924) and an accuracy of 84.16% than the other 4 models (all *p* < 0.05). The predicted probability formula of model 5 was as follows: P(z) = 1/(1 + e^−z^), z = 0.033 × age—0.686 × elevation of C-reactive protein level—1.995 × fever + 0.797 × family history of cancer + 1.459 × air space—0.212 × CT attenuation value. The differences in AUC values among all models are demonstrated in Table 4.

**Table 3:** The diagnostic performances of different models in differentiating pneumonic-type IMA and infectious pneumonia

Model	AUC (95%CI)	Accuracy (%)	Sensitivity (%)	Specificity (%)
Model 1	0.786 (0.739–0.828)	69.79 (0.796–0.699)	83.58 (0.773–0.899)	60.87 (0.542–675)
Model 2	0.679 (0.626–0.728)	68.33 (0.682–0.685)	65.70 (0.576–0.737)	70.00 (0.638–0.763)
Model 3	0.784 (0.737–0.827)	76.25 (0.761–0.764)	64.20 (0.561–0.723)	84.10 (0.791–0.890)
Model 4	0.817 (0.772–0.857)	77.13 (0.770–0.772)	79.10 (0.722–0.860)	75.85 (0.700–0.817)
Model 5	0.893 (0.856–0.924)	84.16 (0.841–0.842)	73.88 (0.664–0.813)	90.82 (0.869–0.948)

Model 1: age, elevation of C-reactive protein level, fever and family history of cancer; Model 2: air space; Model 3: CT attenuation value; Model 4: model 2 + model 3; Model 5: model 1 + model 4

*AUC* area under the curve, *CI* confidence interval

**Table 4:** Comparison of the AUC values between different models in differentiating pneumonic-type IMA and infectious pneumonia

Model	AUC values	*p* value
Model 1 versus model 2	0.786 versus 0.679	0.004
Model 1 versus model 3	0.786 versus 0.784	0.965
Model 1 versus model 4	0.786 versus 0.817	0.397
Model 1 versus model 5	0.786 versus 0.893	< 0.001
Model 2 versus model 3	0.679 versus 0.784	0.003
Model 2 versus model 4	0.679 versus 0.817	< 0.001
Model 2 versus model 5	0.679 versus 0.893	< 0.001
Model 3 versus model 4	0.784 versus 0.817	0.03
Model 3 versus model 5	0.784 versus 0.893	< 0.001
Model 4 versus model 5	0.817 versus 0.893	< 0.001

Model 1: age, elevation of C-reactive protein level, fever and family history of cancer; Model 2: air space; Model 3: CT attenuation value; Model 4: model 2 + model 3; Model 5: model 1 + model 4

*AUC* area under the curve

### Inter-observer agreement

The Cohens kappa coefficient of lesion margin, air bronchogram, interlobular fissure bulging, air space, satellite lesions, pleural effusion, and lymphadenopathy were 0.848 (95CI% 0.767–0.918), 0.921 (95CI% 0.880–0.958), 0.951 (95CI% 0.897–0.989), 0.947 (95CI% 0.911–0.977), 0.966 (95CI% 0.919–0.999), 0.970 (95CI% 0.941–0.994), 0.953 (95CI% 0.918–0.986), respectively, and the intraclass correlation coefficient of CT attenuation value was 0.937 (95CI% 0.923–0.949) for inter-observer agreement.

## Discussion

The treatment of pneumonic-type IMA and infectious pneumonia is totally different [5, 11]. Therefore, it is necessary to distinguish between pneumonic-type IMA and infectious pneumonia. In addition, clinical and radiologic data are considered as valuable tools to differentiate them [7, 12]. In our study we compared the clinical and CT imaging characteristics between the pneumonic-type IMA and infectious pneumonia groups and presented several significant features that can assist to differentiate these lesions.

In view of the overlapping clinical symptoms such as cough and sputum, this pneumonic-type IMA is often misdiagnosed as infectious pneumonia, resulting in a delay in diagnosis and treatment [6]. Consistent with Zong et al. [13], we found that no significant difference was observed in cough and sputum between pneumonic-type IMA and infectious pneumonia (*p* > 0.05). In line with previous studies [14], our study indicated that fever and laboratory results (elevation of white blood cell count and C-reactive protein level) were more commonly associated with infectious pneumonia. In addition, our findings suggested that pneumonic-type IMA was more likely to occur in older people and in families with a history of tumors, which was similar to the results in several studies [15].

Previous studies have assessed the CT imaging features of pneumonic-type lung IMA and proposed the several useful imaging characteristics in identifying the pneumonic-type lung IMA [5, 16]. Consistent with several studies [9, 16], our study confirmed irregular air bronchogram as a key feature in differentiating pneumonic-type IMA from infectious pneumonia. The dilatation, stiffness, or narrowing of bronchi in IMA may be as a result of tumor invasion [17]. However, Jung et al. [8] found that air bronchogram was not helpful in differentiating two groups. We presume that sample size may account for this discrepancy. Our results also indicated that interlobular fissure bulging was more frequently detected in pneumonic-type IMA, which conforms to the findings of several investigators [16]. The interlobular fissure bulging may be caused by increased lung parenchymal volume as a result of mucus secretion by IMA tumor cells [18]. Contrary to the findings of Jung et al. [8], we found the air space was helpful in distinguish two lesions. This is because tumor cells are more likely to invade the bronchus to form a one-way back valve, leading to cavity formation [19]. In our study, we reported that the satellite lesions were more common in pneumonic-type IMA. Not only this, satellite lesions in infectious pneumonia showed frequently ill-defined fibrotic lesions on CT. In addition, we showed that pneumonic-type IMA had lower CT attenuation value than infectious pneumonia, which is consistent with several studies [8, 9]. The lower CT attenuation value was primarily the result of mucus secretion by the IMA tumor.

In this study, we developed the clinical model (model 1) with independent clinical predictors of age, elevation of C-reactive protein level, fever and family history of cancer, with an AUC of 0.786 and an accuracy of 69.79%. And we built the CT imaging model (model 4) with two independent imaging predictors of air space and CT attenuation value, with an AUC of 0.817 and an accuracy of 77.13%. Both models had good diagnostic efficacy and no significant difference in the AUC values between two models (*p* > 0.05). In addition, we developed the combined model (model 5) by incorporating all independent clinical and radiologic predictors, with an AUC of 0.893 and an accuracy of 84.16%. And the AUC value of the combined model was significantly higher than that of the clinical factor model (*p* < 0.001) and CT imaging model (*p* < 0.001). Therefore, we demonstrated that the evaluation of clinical and CT imaging features can assist in differentiating between pneumonic-type IMA and infectious pneumonia.

Given the low incidence of IMA, most studies about pneumonic-type IMA were with small samples and without sufficient clinical information [8, 9, 17]. Therefore, we collected detailed clinical and radiologic data from 6 situations for research. Beck et al. [20] proposed that IMA can show spontaneous regression of airspace opacities on CT without anticancer drugs and explained that it was due to mucus flow or combined inflammation. In our study, all lung IMA were pathologically confirmed. In addition, the evidence of pneumonia is either pathological confirmation due to misdiagnosis as lung cancer or disappearance of lesions on follow-up CT after anti-inflammatory therapy. And the complete disappearance of pneumonia lesions on follow-up CT after anti-inflammatory treatment suggested no cases with combined tumors.

There are several limitations in our study. First, this is a retrospective study and is subject to inherent limitations associated with retrospective analyses. Second, CT imaging was acquired from different scanners at six centers. However, their CT protocol was similar. In particular, tube voltage, a major parameter affecting CT values, was consistent. Third, given the difficulty of collecting cases, other pathological types of lung cancer with consolidation mimicking pneumonia on CT were not studied, future work should further investigate other lung cancers. Forth, we selected to evaluate the imaging features on non-enhanced CT rather than on contrast-enhanced CT. Although the contrast-enhanced CT imaging can provide more information to differentiate pneumonia-type IMA from pneumonia, plain scan has no contraindications to contrast administration in the clinical practice [21, 22]. And we have obtained relatively good diagnostic efficacy by image features on non-enhanced CT in this study.

In conclusion, our findings showed that pneumonic-type IMA and infectious pneumonia have different clinical and imaging characteristics. Detailed evaluation of clinical and CT imaging characteristics is useful for differentiating pneumonic-type IMA and infectious pneumonia.

## Data Availability

The data used and analyzed during the current study are available from the corresponding author on reasonable request.

## Abbreviations

AUC: Area under the curve
CI: Confidence interval
IMA: Invasive mucinous adenocarcinoma
OR: Odds ratio
ROC: Receiver operating characteristic
ROI: Regions of interest

## References

[1] Ferlay J, Colombet M, Soerjomataram I (2019). Estimating the global cancer incidence and mortality in 2018: GLOBOCAN sources and methods. Int J Cancer.

[2] Goldstraw P, Ball D, Jett JR (2011). Non-small-cell lung cancer. Lancet.

[3] Nakagomi T, Goto T, Hirotsu Y (2018). Genomic characteristics of invasive mucinous adenocarcinomas of the lung and potential therapeutic targets of B7–H3. Cancers (Basel).

[4] Shang G, Jin Y, Zheng Q (2019). Histology and oncogenic driver alterations of lung adenocarcinoma in Chinese. Am J Cancer Res.

[5] Wang T, Yang Y, Liu X (2021). Primary invasive mucinous adenocarcinoma of the lung: prognostic value of CT imaging features combined with clinical factors. Korean J Radiol.

[6] Pascoe HM, Knipe HC, Pascoe D, Heinze SB (2018). The many faces of lung adenocarcinoma: a pictorial essay. J Med Imaging Radiat Oncol.

[7] Nie K, Nie W, Zhang YX, Yu H (2019). Comparing clinicopathological features and prognosis of primary pulmonary invasive mucinous adenocarcinoma based on computed tomography findings. Cancer Imaging.

[8] Jung JI, Kim H, Park SH (2001). CT differentiation of pneumonic-type bronchioloalveolar cell carcinoma and infectious pneumonia. Br J Radiol.

[9] Kim TH, Kim SJ, Ryu YH (2006). Differential CT features of infectious pneumonia versus bronchioloalveolar carcinoma (BAC) mimicking pneumonia. Eur Radiol.

[10] Yoshizawa A, Motoi N, Riely GJ (2011). Impact of proposed IASLC/ATS/ERS classification of lung adenocarcinoma: prognostic subgroups and implications for further revision of staging based on analysis of 514 stage I cases. Mod Pathol.

[11] Horiguchi T, Yanagi S, Tomita M (2021). A case of bilateral invasive mucinous adenocarcinoma of the lung with severe productive cough and dyspnea successfully treated with palliative lung lobectomy. Respir Med Case Rep.

[12] Watanabe H, Saito H, Yokose T (2015). Relation between thin-section computed tomography and clinical findings of mucinous adenocarcinoma. Ann Thorac Surg.

[13] Zong Q, Zhu F, Wu S (2021). Advanced pneumonic type of lung adenocarcinoma: survival predictors and treatment efficacy of the tumor. Tumori.

[14] Yao Z, Zhang Y, Wu H (2019). Regulation of C-reactive protein conformation in inflammation. Inflamm Res.

[15] Shim HS, Kenudson M, Zheng Z (2015). Unique genetic and survival characteristics of invasive mucinous adenocarcinoma of the lung. J Thorac Oncol.

[16] Huo JW, Huang XT, Li X, Gong JW, Luo TY, Li Q (2021). Pneumonic-type lung adenocarcinoma with different ranges exhibiting different clinical, imaging, and pathological characteristics. Insights Imaging.

[17] Akira M, Atagi S, Kawahara M, Iuchi K, Johkoh T (1999). High-resolution CT findings of diffuse bronchioloalveolar carcinoma in 38 patients. AJR Am J Roentgenol.

[18] Han J, Wu C, Wu Y (2021). Comparative study of imaging and pathological evaluation of pneumonic mucinous adenocarcinoma. Oncol Lett.

[19] Tan Y, Gao J, Wu C (2019). CT characteristics and pathologic basis of solitary cystic lung cancer. Radiology.

[20] Beck KS, Sung YE, Lee KY, Han DH (2020). Invasive mucinous adenocarcinoma of the lung: serial CT findings, clinical features, and treatment and survival outcomes. Thorac Cancer.

[21] Oudkerk M, Liu S, Heuvelmans MA, Walter JE, Field JK (2021). Lung cancer LDCT screening and mortality reduction—evidence, pitfalls and future perspectives. Nat Rev Clin Oncol.

[22] Succony L, Rassl DM, Barker AP, McCaughan FM, Rintoul RC (2021). Adenocarcinoma spectrum lesions of the lung: detection, pathology and treatment strategies. Cancer Treat Rev.

